# Identifying Alzheimer’s disease-related proteins by LRRGD

**DOI:** 10.1186/s12859-019-3124-7

**Published:** 2019-11-25

**Authors:** Tianyi Zhao, Yang Hu, Tianyi Zang, Liang Cheng

**Affiliations:** 1 School of Life Science and Technology, Department of Computer Science and Technology, Harbin Institute of Technology, Harbin, China; 2 College of Bioinformatics Science and Technology, Harbin Medical University, Harbin, 150001 China

**Keywords:** Alzheimer’s disease, Proteins, Similarity of diseases, Logistic regression, Gradient descent

## Abstract

**Background:**

Alzheimer’s disease (AD) imposes a heavy burden on society and every family. Therefore, diagnosing AD in advance and discovering new drug targets are crucial, while these could be achieved by identifying AD-related proteins. The time-consuming and money-costing biological experiment makes researchers turn to develop more advanced algorithms to identify AD-related proteins.

**Results:**

Firstly, we proposed a hypothesis “similar diseases share similar related proteins”. Therefore, five similarity calculation methods are introduced to find out others diseases which are similar to AD. Then, these diseases’ related proteins could be obtained by public data set. Finally, these proteins are features of each disease and could be used to map their similarity to AD. We developed a novel method ‘LRRGD’ which combines Logistic Regression (LR) and Gradient Descent (GD) and borrows the idea of Random Forest (RF). LR is introduced to regress features to similarities. Borrowing the idea of RF, hundreds of LR models have been built by randomly selecting 40 features (proteins) each time. Here, GD is introduced to find out the optimal result. To avoid the drawback of local optimal solution, a good initial value is selected by some known AD-related proteins. Finally, 376 proteins are found to be related to AD.

**Conclusion:**

Three hundred eight of three hundred seventy-six proteins are the novel proteins. Three case studies are done to prove our method’s effectiveness. These 308 proteins could give researchers a basis to do biological experiments to help treatment and diagnostic AD.

## Background

Alzheimer’s disease [1] has become the greatest threat to the elderly. At present, there is no effective drug for AD. Many studies have reported that neurodegenerative diseases such as Alzheimer’s disease are closely related to aging diseases and can interact with each other [2, 3]. Many scholars reported that abnormal behavior of specific proteins is the key to cause AD [4, 5]. This is because the main pathological feature of AD patients is that a large number of beta amyloid (A beta) deposits are formed outside the neurons in the cortex and hippocampus and neurofibrillary tangles (NFT) are formed in neurons with tau protein as the main component [6, 7].

Recently, finding alternatives to diagnosing AD has become a hot issue [8]. Ray et al. found 18 plasma proteins have high specificity in AD patients. They then found that these proteins were associated with Aβ and tau levels in CSF. Then the Human Discovery Multi-Analyte Profile (MAP) has become a popular tool to identify plasma analytes. But, these exciting results raise a major issue that it is hard to reproduce these protein panels [8]. Gisslen M et al. [9] found that the correlation between CSF and plasma NFL was stronger than tau. Olsson B et al. [10] confirmed this view, and they found that the NFL was increasing in both AD patients and MCI’s CSF. Studies have found this phenomenon in serum and plasma samples as well [11]. O’Bryant et al. [12] used a serum-based algorithm to distinguish AD from Parkinson’s disease and cross-validated this algorithm. At present, biological experiments and bioinformatics methods are the most widely used methods. Lista et al. [13] reviewed the blood biomarkers of AD disease based on mass spectrometry. They concluded that about 20 proteins may be potential biomarkers of AD diseases. They also emphasized that the molecular level of neurodegenerative diseases (such as AD) may change 20 years before the onset of clinical symptoms.

Complex protein interactions could be researched by Protein-protein interaction (PPI) network [2, 14, 15]. Most PPI networks are built based on genes’ relationship. Shubhabrata et al. [16] used dense module searching (DMS) method to integrate gene-wide association results into PPI network and identified candidate genes or sub-networks for AD. However, most of protein networks are static network which has highly average and idealized network structures. In fact, with the change of external conditions, some proteins will be degraded, while others will be translated [17]. This would result in the new protein interactions and disappearance of old protein interactions.

Based on the prior knowledge of protein interaction and biology, some researchers use machine learning [17, 18] and pattern classification methods [19] to predict diseases-related protein interaction. Machine learning methods include Bayesian network method [20], Markov model method [21], Random Forest method [22] and Support Vector Machine method [23] etc. Barber et al. [24] uses Simulated Annealing (SA) to select the proteins most relevant to AD and uses Random Forest (RF) to classify patients based on these proteins. The best model trained in serum can significantly predict disease status with AUC of 0.66. At the same time, training with serum data and testing by CSF data, the AUC is 0.77. However, machine learning method usually needs negative samples, but in fact, negative samples are hard to obtain.

Therefore, in this paper, we consider the problem of identifying AD-related proteins as a regression problem, which makes it unnecessary for us to obtain negative sets. This can greatly improve the accuracy of recognition and reduce the false positive rate.

## Methods

### Data collection and database content

#### Disease ontology

Three thousand five hundred twenty-four kinds of diseases are downloaded from Disease Ontology (DO) which is an authoritative website that contains comprehensive disease related knowledge [25]. The concept of each disease or disease is a node in DO. Each node has an ID. There is a subordinate relationship between nodes. Similarity between AD and other diseases could be obtained based on DO using similarity calculation methods.

#### Uniprot

UniProt [26] consists of three parts: UniProt Knowledgebase (UniProt), which is the information access center of protein sequence, function, classification, cross-reference, etc. UniProt Non-redundant Reference (UniRef) database, which combines closely related protein sequences into a single record to improve search speed; currently, three sub-libraries are formed according to sequence similarity, namely UniRef100, UniRef90 and UniRef50; UniProt Archive (UniParc) is a repository that records the history of all protein sequences. Users can query database by text, search database by BLAST program, or download data directly by FTP. All known diseases-related proteins could be obtained by UniProt.

#### Gene ontology

Gene ontology (GO) is one of the most successful ontology in the field of biomedicine. It provides a standard and accurate term set for describing the molecular function, biological process and other related information, which is widely used in the field of biomedical research.

### Disease similarity

Firstly, similarity between AD and other diseases are calculated by five methods. At present, these five methods are widely used: Resnik’s [27], Lin’s [28], Wang’s [5], Process-similarity Based (PSB) [29], SemFunSim [30].

The principle of Resnik’s method and Lin’s method is same. Both of them calculate similarity by GO terms, but Resnik’s method uses the information content (IC) of the most informative common ancestor (MICA) between two terms. However, Wang’s method improves Resnik’s method. It considers multiple common ancestors. PSB: associations of GO terms are considered. Semfensim: semantic and gene functional association are intergrated to calculate similarity. Since it is hard to recognize which method is the best, all of them are used to calculate similarities. Finally, 3524 diseases’ similarity with AD are calculated. Therefore, each disease gets 5 different similarity values, and we add these five values together as the final similarity.

**Fig. 1:**
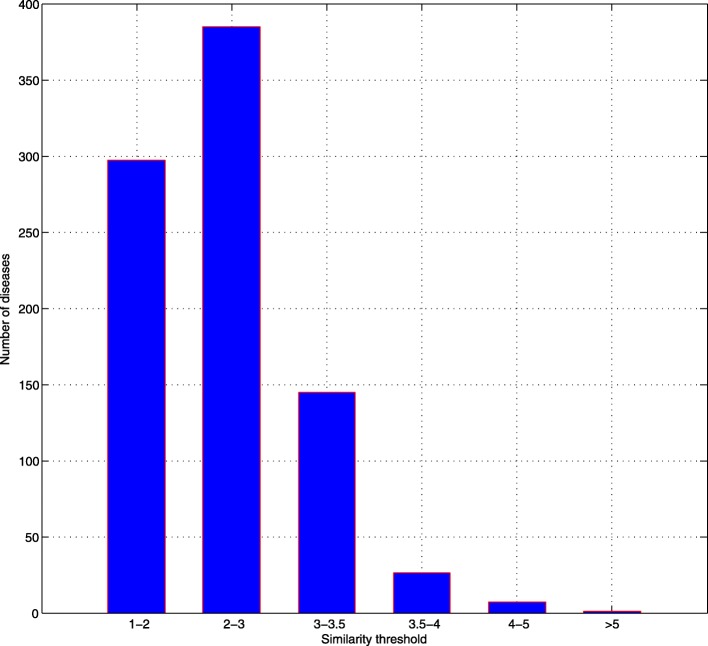
Probability distribution of disease similarity

Figure 1 shows all the similarities which are higher than 1 between 3524 diseases and AD. Two thousand six hundred sixty-three of three thousand five hundred twenty-four diseases’ similarity is lower than 1, so they did not show in the Fig. 1. As we can see, since 99% diseases’ similarities are less than 3.5, 3.5 is set as a threshold to retain only a small number of diseases most associated with AD.

Finally, there are 34 diseases left. Table 1 shows their similarity with AD and the names of them.

**Table 1:** Similarities between AD and other diseases by five different methods

DOID	SemFunSim	Wang	Lin	PSB	Resnik	Total
0050784	0.02	0.48	0.40	0.06	2.55	3.52
0060368	0.01	0.48	0.42	0.06	2.55	3.52
0050765	0.00	0.63	0.33	0.00	2.55	3.52
14,784	0.00	0.63	0.33	0.00	2.55	3.52
1440	0.02	0.48	0.40	0.08	2.55	3.52
12,705	0.01	0.48	0.39	0.10	2.55	3.53
936	0.23	0.53	0.62	0.09	2.09	3.56
13,548	0.00	0.63	0.35	0.02	2.55	3.57
3981	0.00	0.63	0.35	0.03	2.55	3.57
4873	0.01	0.48	0.39	0.14	2.55	3.57
9277	0.01	0.63	0.38	0.00	2.55	3.58
0060264	0.01	0.63	0.39	0.00	2.55	3.59
12,704	0.03	0.48	0.44	0.08	2.55	3.59
1441	0.04	0.54	0.48	0.00	2.55	3.63
12,377	0.04	0.54	0.47	0.02	2.55	3.63
0050950	0.05	0.54	0.48	0.00	2.55	3.63
14,332	0.00	0.54	0.30	0.28	2.55	3.68
4752	0.03	0.54	0.44	0.12	2.55	3.69
2378	0.04	0.48	0.45	0.22	2.55	3.75
0050968	0.00	0.48	0.30	0.44	2.55	3.77
0050951	0.08	0.63	0.53	0.00	2.55	3.80
12,217	0.06	0.44	0.47	0.31	2.55	3.84
230	0.11	0.54	0.55	0.15	2.55	3.91
12,858	0.09	0.63	0.52	0.12	2.55	3.91
331	0.46	0.65	0.73	0.02	2.09	3.95
332	0.14	0.54	0.58	0.13	2.55	3.95
11,870	0.03	0.63	0.41	0.38	2.55	4.00
0050890	0.21	0.63	0.63	0.00	2.55	4.03
3213	0.19	0.63	0.65	0.10	2.55	4.12
2377	0.19	0.54	0.64	0.19	2.55	4.12
231	0.15	0.63	0.60	0.19	2.55	4.13
14,330	0.19	0.54	0.62	0.24	2.55	4.16
1289	0.57	0.75	0.83	0.27	2.55	4.98
680	1.00	0.87	1.00	0.00	3.60	6.47

### Extracting features

Firstly, the 34 disease’s name are obtained by the ID of DO. Then, we obtained 34 disease-related proteins on the Uniprot. To ensure the accuracy of the results, only human and reviewed proteins are selected.

We excluded two disease: DOID: 936 ‘brain disease’ and DOID: 14332 ‘postencephalitic Parkinson disease’. Brain disease is related to more than 2000 proteins and it is a large group of diseases and includes AD. postencephalitic Parkinson disease has no related information in Uniprot, so we removed this disease from data too. Therefore, 32 diseases are left and we obtained 32 diseases-related proteins by Uniprot.

**Fig. 2:**
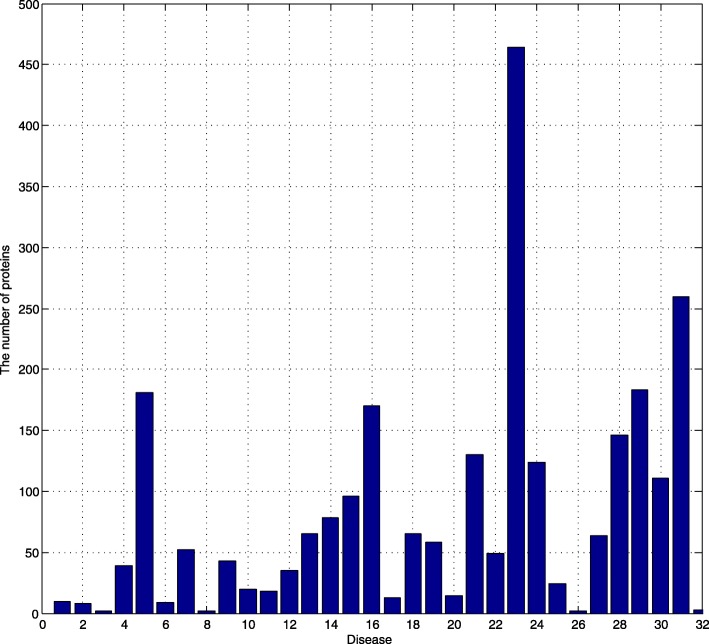
Number of proteins corresponding to each disease

Figure 2 shows the number of proteins for each protein. AD is related to 299 proteins. Therefore, 33 kinds of diseases are related to 2827 proteins. Some of the 2827 proteins are duplicated, which indicates that similar diseases share similar proteins. Firstly, we removed the redundant proteins and 1608 kinds of proteins are left. To our surprise, 43.1% proteins are redundant. So there must be some AD-related proteins that we have not known that they are related to AD, but we have known that they act on AD’s similar diseases.

As we mentioned before, proteins are the features for similarity. Therefore, the dimension of feature’s matrix is 1608. Each disease corresponds to a 1608*1 feature matrix.

Each protein has a weight for similarity and it represents its relationship with AD. Constantly iterating over these weights so that they can map to similarities and get their relationship with AD.

### Map features to similarity by logistics regression

Firstly, we normalized all diseases’ similarity. All similarities are transformed into a number between 0 and 1. The similarity between AD and AD itself should be the max number in all methods. For Resnik method, the max number is 4 and other methods are 1. Therefore, the max similarity is 8. Then we could normalize all other diseases’ similarity by eq. (1).

1 $$ {similarity}_{normalized}= similarity/8 $$

Thirty-two diseases are 32 samples and 1608 proteins are 1608 dimensions of feature. It is a typical high dimension and small samples problem. LR could hardly solve this problem. Therefore, we borrowed the idea of Random Forests (RF). Forty features (proteins) are randomly selected to build model each time. The 40 features (proteins) would be put back after building model. We selected 40 features because $\sqrt{1608}\approx 40$. This is the typical way to select the number of features in RF. We would repeat 400 times so that each protein would be selected nearly 10 times.

After building models every time, GD is used to find out optimal result. Since GD is easy to get local optimal solution rather than global optimal solution, we used the known AD-related protein as the initial value of the iteration. In this way, the initial value is very close to the global optimal solution so we can get the global optimal solution with fewer iterations.

**Fig. 3:**
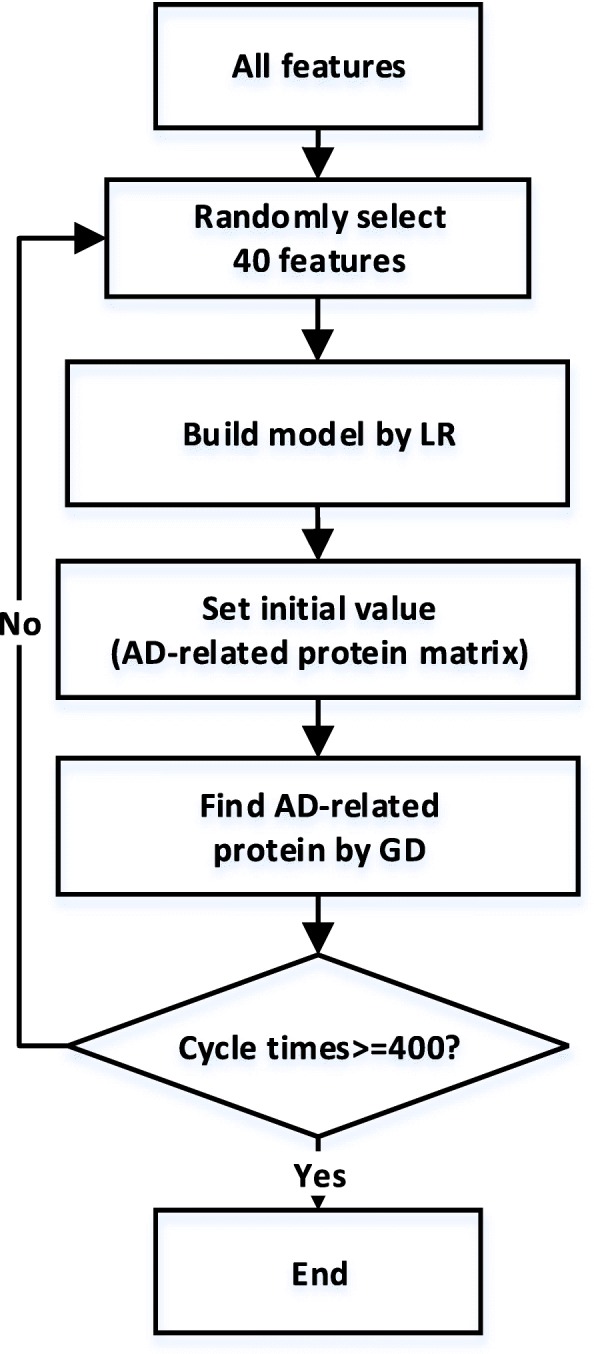
The work flow of selecting features and building mode

Figure 3 shows the work flow of selecting features and building models.

The workflow of LR is shown in Table 2.

**Table 2:** Work flow of LR

Work flow of LR	
Step 1. Constructing a prediction function	
$$ {h}_{\theta }(x)=g\left({\theta}^Tx\right)=\frac{1}{1+{e}^{-{\theta}^Tx}} $$	
*θ*is regression variable, x is independent variable	
Step 2. Construction loss function	
$$ J\left(\theta \right)=-\frac{1}{m}\sum \limits_{i=1}^m\left[{y}_i\log {h}_{\theta}\left({x}_i\right)+\left(1-{y}_i\right)\log \left(1-{h}_{\theta}\left({x}_i\right)\right)\right] $$	
y is true similarity, m is the number of sample	
Step 3. Newton method for getting the minimum *J*(*θ*)	
$$ \theta \leftarrow \theta -\frac{l^{\hbox{'}}\left(\theta \right)}{l^{\hbox{'}\hbox{'}}\left(\theta \right)} $$	
*l*(*θ*) is maximum likelihood function	

Through the above steps, we can build a logistic regression function: ${h}_{\theta }(x)=\frac{1}{1+{e}^{-{\theta}^Tx}}$. X which is our input is 1608 proteins for each disease, the output h(x) is the similarity between each disease and AD.

Obviously, the similarity between disease and AD is not the result we hope to obtain. So if we can find a suitable weight for each protein, the similarity between AD and AD itself would be 1. Then, the weight is reasonable and we can obtain the AD-related protein by these weights.

### Find AD-related proteins by gradient descent

Therefore, Gradient Descent (GD) is introduced to solve the model obtained by LR.

GD is a kind of optimization method. The work flow of GD is shown in Table 3.

**Table 3:** Work flow of GD

Work flow of GD	
Step 1. Finding descent direction	
$$ \nabla =\frac{\partial f}{\partial x} $$	
Step 2. Moving x	
*x* = *x* − *k*∇	
k is descent rate.	
Step 3. Repeat step 2, until satisfied with the following equation	
*f*(*x*_*n* + 1_) − *f*(*x*_*n*_) < *ε*	
*ε* is any constant.	

Through the above steps, feature matrix of AD-related proteins are obtained. The 1 in matrix represents that this protein is related to AD.

**Fig. 4:**
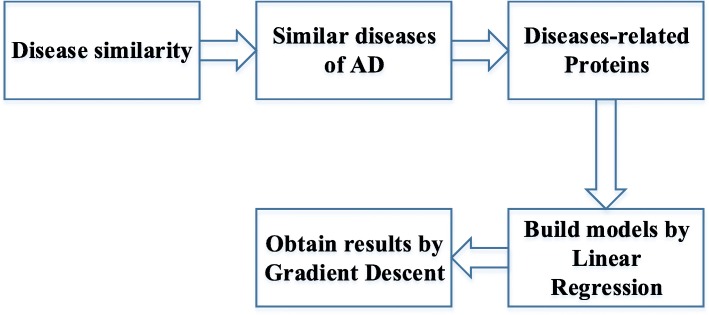
The work flow of selecting features and building models

Figure 4 shows our workflow. Firstly, the similarity between AD and other diseases could be calculated. Then We can get diseases similar to AD. In addition, these diseases-related proteins could be obtained by Uniprot. Finally, LR could be used to build models. After that, GD should be used to obtain the optimal results.

## Results

A. Data process
B. Result

Since 400 models are built by LR, 400 kinds of results are obtained. Each protein has 10 times chances to be selected as features and algorithm can judge whether it is related to AD. Therefore, the maximum number of times for each protein to be related to AD is 10, and the minimum number is 0.

**Fig. 5:**
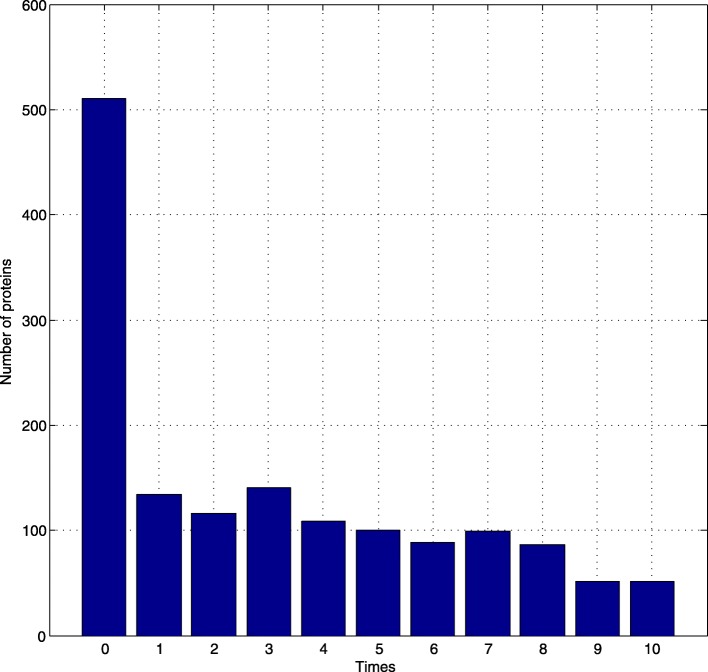
Times that proteins are thought to be related to AD

Figure 5 shows the times that proteins are thought to be related to AD.

As we can see in Fig. 5, more than 500 kinds of proteins are unrelated to AD. Algorithm never gets results that they are AD-related proteins. However, about 50 kinds of proteins are identified to be related to AD for 10 times.

Seven times is set as a threshold to select AD-related proteins. If proteins are thought to be related to AD more than 7 times by algorithm, the proteins are related to AD. Otherwise, we did not consider them as AD-related proteins. There are 376 such proteins.

**Fig. 6:**
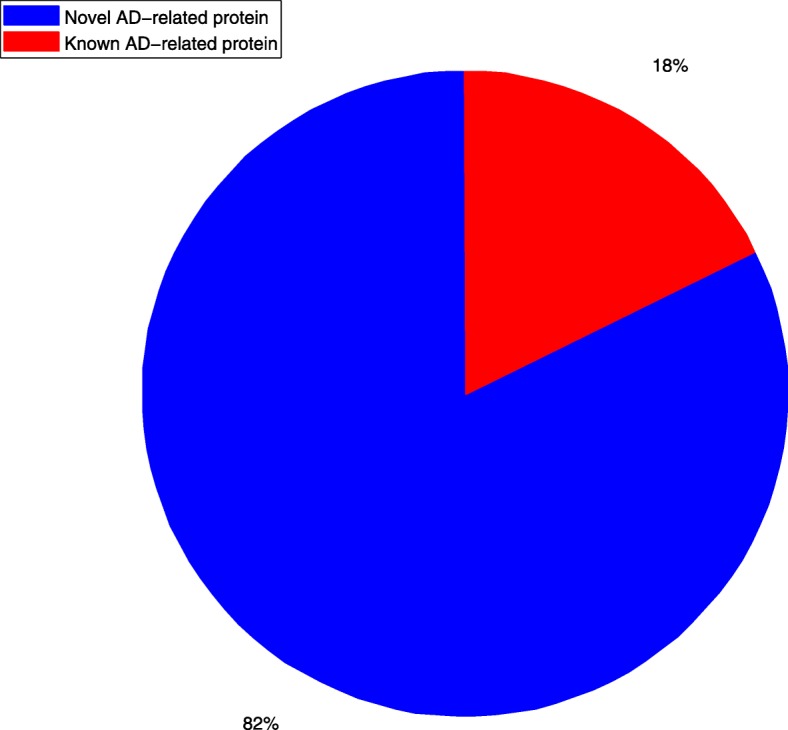
The proportion of known AD-related proteins to novel AD-related proteins

The Fig. 6 shows the proportion of newly discovered proteins and known proteins.

As we can see, 18% of 376 proteins are known AD-related proteins. Most of proteins are associated with AD-like diseases and researchers do not know that they are associated with AD.

C. Case study

Three case studies are done to verify our method’s effectiveness. We selected three novel proteins from 308 novel AD-related proteins.

1. SUMO-conjugating enzyme UBC9
  In UniProt, there is no information about the relationship between this protein and AD. Our method identifies the strong correlation between AD and AD. (10 times). Several research have found that UBC9 plays an important role in AD due to its function is associated with the aggregation of beta-amyloid protein (Aβ). It can interact with target protein and change their localization, activity, or stability. LE Mcmillan et al. [31] demonstrated this in 2011.
2. Kinesin light chain 2 (KLC2)
  APP is known important gene to AD. KLC2 can interact with APP and it is considered to be related to AD. Kamal et al. [32] reported that KLC2 can affect transport of APP into axons. S Matsuda et al. ‘s study [33] also demonstrated that KLC2 causes AD by affecting APP.
3. Kinesin heavy chain isoform 5C (KIF5C)
  KIF5A showed pan-neuronal distribution in the nervous system. KIF5B plays an important roles in the maintenance of motor neurons rather than in their formation. D Sepulvedafalla et al. [34] found that KIF5C are highly related to familial AD and neurodegeneration.

## Discussion

Identifying the AD-related proteins can help us treatment and diagnose AD better. It saves lots of researchers’ time and money. Doing biological experiments by the priority is an efficient way to understand the mechanism of AD.

Here we purposed a method to identify the AD-related proteins based on a hypothesis which is similar disease share similar proteins. Here is no doubt that proteins have contribution to the similarity of symptoms between diseases.

Therefore, the first step is to calculate the similarity between other diseases and AD. We totally used 5 methods to obtain the similarity. 3.5 was set as threshold to screen diseases which are most related to AD. There are 34 diseases left. Then, we downloaded these diseases-related proteins by Uniprot. Due to the reason mentioned in method section C, 2 diseases are excluded.

Then we aggregate the proteins that correspond to these diseases. Each protein is a one-dimensional feature, and we try to map these features to similarity. Because this is a small sample of high-dimensional problems, the use of LR alone is not enough to solve this problem. Here, we borrowed the idea of RF: randomly selected 40 features to build model by LR each time. Then, GD is introduced to find out the optimal result. After 400 models are built, we summarized the whole results and set 7 as threshold to screen the AD-related proteins.

Finally, we obtained 376 proteins which are related to AD. Three hundred eight of three hundred seventy-six proteins are novel. We selected three of them to do case studies to prove our method’s effectiveness.

## Conclusions

Identification of disease-related proteins is essential for developing new drugs and understanding the pathogenesis. In view of the shortcomings of current machine learning methods and protein interaction networks, we propose a regression method, which can effectively avoid the shortcomings of obtaining negative samples and the inability of the network to change dynamically. It provides a new way to solve disease-related proteins, that is, to transform classification or clustering problems into regression problems.

This paper proposes a hypothesis that similar diseases share similar proteins. A total of 2827 proteins were obtained by searching 32 disease-related proteins in Uniprot, but they are only 1608 kinds of proteins, which shows that this hypothesis is valid. Similar diseases have multiple protein duplications.

In the aspect of algorithm innovation, we combine LR with RF to solve the problem of small sample and high dimension. In order to overcome the problem that GD often falls into local optimum, we get a very reasonable initial iteration value.

The results show that this method has certain practical value and is helpful for further research. Through our method, we can find more disease-related proteins.

## Data Availability

All the datasets used in this paper could be downloaded from website.

## Abbreviations

AD: Alzheimer’s disease
DO: Disease Ontology
GD: Gradient Descent
GO: Gene Ontology
IC: Information Content
KIF5C: Kinesin heavy chain isoform 5C
LR: Logistic Regression
MICA: Most Informative Common Ancestor
NFT: Neurofibrillary Tangles
PPI: Protein-protein interaction
PSB: Process-similarity Based
RF: Random Forests
SUMO: Small Ubiquitin-related Modifier

## References

[1] Cummings J, Lee G, Mortsdorf T, Ritter A, Zhong K (2017). Alzheimer’s disease drug development pipeline: 2017. Alzheimers Dement Transl Res Clin Interv.

[2] Peng J, Guan J, Shang X (2019). Predicting Parkinson's disease genes based on node2vec and autoencoder. Front Genet.

[3] Hu Y, Zhao T, Zang T, Zhang Y, Cheng L. Identification of Alzheimer’s disease-related genes based on data integration method. Front Genet. 2018;9:730. 10.3389/fgene.2018.00703 PMC6355707 30740125

[4] Jellinger KA (2003). General aspects of neurodegeneration. J neural Transm Suppl. J Neural Transm Suppl.

[5] Wang JZ, Zhidian D, Rapeeporn P, Yu PS, Chin-Fu C (2007). A new method to measure the semantic similarity of GO terms. Bioinformatics.

[6] Navarromabarak C, Camachocarranza R, Espinosaaguirre JJ (2018). Cytochrome P450 in the central nervous system as a therapeutic target in neurodegenerative diseases. Drug Metab Rev.

[7] Leon MJD, Convit A, Wolf OT, Tarshish CY, Desanti S, Rusinek H, Tsui W, Kandil E, Scherer AJ, Roche A (2001). Prediction of cognitive decline in normal elderly subjects with 2-[18F]fluoro-2-deoxy-d-glucose/positron-emission tomography (FDG/PET). Proc Natl Acad Sci U S A.

[8] Henriksen K, O’Bryant SE, Hampel H, Trojanowski JQ, Montine TJ, Jeromin A, Blennow K, Lönneborg A, Wyss-Coray T, Soares H (2014). The future of blood-based biomarkers for Alzheimer's disease. Alzheimers Dement.

[9] Zetterberg H, Wilson D, Andreasson U, Minthon L, Blennow K, Randall J, Hansson O (2013). Plasma tau levels in Alzheimer's disease. Alzheimers Res Ther.

[10] Olsson B, Lautner R, Andreasson U, Öhrfelt A, Portelius E, Bjerke M, Hölttä M, Rosén C, Olsson C, Strobel G (2016). CSF and blood biomarkers for the diagnosis of Alzheimer's disease: a systematic review and meta-analysis. Lancet Neurol.

[11] Bacioglu M, Maia LF, Preische O, Schelle J, Apel A, Kaeser SA, Schweighauser M, Eninger T, Lambert M, Pilotto A (2016). Neurofilament light chain in blood and CSF as marker of disease progression in mouse models and in neurodegenerative diseases. Neuron.

[12] O'Bryant SE, Xiao G, Zhang F, Edwards M, German DC, Yin X, Como T, Reisch J, Huebinger RM, Graff-Radford N (2014). Validation of a serum screen for Alzheimer's disease across assay platforms, species, and tissues. J Alzheimers Dis.

[13] Lista S, Dubois B, Hampel H (2015). Paths to Alzheimer’s disease prevention: from modifiable risk factors to biomarker enrichment strategies. J Nutr Health Aging.

[14] Peng J, Wang Y, Jin C (2014). Towards integrative gene functional similarity measurement. BMC Bioinformatics.

[15] Peng J, Wang X, Shang X (2019). Combining gene ontology with deep neural networks to enhance the clustering of single cell RNA-Seq data. BMC Bioinformatics.

[16] Mukherjee S, Kaeberlein M, Kauwe J, Naj AC, Crane P (2014). A systems-biology approach to identify candidate genes for Alzheimer's disease by integrating protein-protein interaction network and subsequent in vivo validation of candidate genes using a C. elegans model of ab toxicity. Alzheimers Dement.

[17] Peng J, Hui W, Li Q, Chen B, Hao J, Jiang Q, Shang X, Wei Z. A learning-based framework for miRNA-disease association identification using neural networks. Bioinformatics. 2019;21(1):4364-71. 10.1093/bioinformatics/btz254 30977780

[18] Cheng L, Hu Y, Sun J, Zhou M, Jiang Q (2018). DincRNA: a comprehensive web-based bioinformatics toolkit for exploring disease associations and ncRNA function. Bioinformatics.

[19] Cheng L, Wang P, Tian R, Wang S, Guo Q, Luo M, Zhou W, Liu G, Jiang H, Jiang Q (2018). LncRNA2Target v2. 0: a comprehensive database for target genes of lncRNAs in human and mouse. Nucleic Acids Res.

[20] Fu C, Deng S, Jin G, Wang X, Yu Z-G (2017). Bayesian network model for identification of pathways by integrating protein interaction with genetic interaction data. BMC Syst Biol.

[21] Krejci A, Hupp TR, Lexa M, Vojtesek B, Muller P (2015). Hammock: a hidden Markov model-based peptide clustering algorithm to identify protein-interaction consensus motifs in large datasets. Bioinformatics.

[22] Xu L, Liao C, Chen G-D, Chang C-C (2019). k-skip-n-gram-RF: a random Forest based method for Alzheimer’s disease protein identification. Front Genet.

[23] Cui Y, Cai M, Stanley HE (2018). Discovering disease-associated genes in weighted protein–protein interaction networks. Physica A Stat Mech Appl.

[24] Barber IS, Nevado-Holgado AJ, Lovestone S (2017). A Parkinson’s disease protein biomarker panel using the Somamer assay and machine learning. Alzheimers Dement.

[25] Schriml LM, Arze C, Nadendla S, Chang YWW, Mazaitis M, Felix V, Feng G, Kibbe WA (2012). Disease ontology: a backbone for disease semantic integration. Nucleic Acids Res.

[26] Consortium UP (2015). UniProt: a hub for protein information. Nucleic Acids Res.

[27] Resnik P (1999). Using information content to evaluate semantic similarity in a taxonomy.

[28] Lin D (1998). An information-theoretic De nition of similarity.

[29] Mathur S, Dinakarpandian D (2012). Finding disease similarity based on implicit semantic similarity. J Biomed Inform.

[30] Cheng L, Li J, Ju P, Peng J, Wang Y (2014). SemFunSim: a new method for measuring disease similarity by integrating semantic and gene functional association. PLoS One.

[31] Mcmillan LE, Brown JT, Henley JM, Cimarosti H (2011). Profiles of SUMO and ubiquitin conjugation in an Alzheimer's disease model. Neurosci Lett.

[32] Kamal A, Stokin GB, Yang Z, Xia CH, Goldstein LSB (2000). Axonal transport of amyloid precursor protein is mediated by direct binding to the Kinesin light chain subunit of Kinesin-I. Neuron.

[33] Matsuda S, Matsuda Y, D'Adamio L (2003). Amyloid beta protein precursor (AbetaPP), but not AbetaPP-like protein 2, is bridged to the kinesin light chain by the scaffold protein JNK-interacting protein 1. J Biol Chem.

[34] Sepulvedafalla D, Barreraocampo A, Hagel C, Korwitz A, Vinuezaveloz MF, Zhou K, Schonewille M, Zhou H, Velazquezperez L, Rodriguezlabrada R (2014). Familial Alzheimer’s disease–associated presenilin-1 alters cerebellar activity and calcium homeostasis. J Clin Investig.

